# Symbiosis of soybean with nitrogen fixing bacteria affected by root lesion nematodes in a density-dependent manner

**DOI:** 10.1038/s41598-020-58546-x

**Published:** 2020-01-31

**Authors:** Ahmed Elhady, Johannes Hallmann, Holger Heuer

**Affiliations:** 1 Department of Epidemiology and Pathogen Diagnostics, Julius Kühn-Institut, Federal Research Centre for Cultivated Plants, Braunschweig, Germany; 2 Department of Plant Protection, Faculty of Agriculture, Benha University, Benha, Egypt

**Keywords:** Parasitism, Rhizobial symbiosis

## Abstract

Early maturing varieties of soybean have a high yield potential in Europe, where the main biotic threat to soybean cultivation are root lesion nematodes (*Pratylenchus* spp.). Nitrogen fixation in root nodules by highly efficient inoculants of *Bradyrhizobium japonicum* is an incentive to grow soybean in low-input rotation systems. We investigated density-dependent effects of *Pratylenchus penetrans* on nitrogen fixation by co-inoculated *B. japonicum*. Less than 130 inoculated nematodes affected the number and weight of nodules, the density of viable bacteroids in nodules, and nitrogen fixation measured as concentration of ureides in leaves. With more inoculated nematodes, the percentage that invaded the roots increased, and adverse effects on the symbiosis accelerated, leading to non-functional nodules at 4,000 and more nematodes. When *P. penetrans* invaded roots that had fully established nodules, growth of nodules, density of bacteroids, and nitrogen fixation were affected but not the number of nodules. In contrast, nodulation of already infested roots resulted in a high number of small nodules with decreased densities of bacteroids and nitrogen fixation. *P. penetrans* invaded and damaged the nodules locally, but they also significantly affected the nodule symbiosis by a plant-mediated mechanism, as shown in an experiment with split-root systems.

## Introduction

Soybean (*Glycine max* (L.) Merrill) is among the economically most important crops worldwide. Its production area is currently increasing in temperate regions. In Germany, the production increased from 1,000 ha in 2003 to 23,900 ha in 2018. On 343,000 ha within Germany, the conditions would allow a potential yield of 3.2 t/ha when growing adapted early maturing varieties^1,2^. One of the intentions of farmers to grow soybean is to diversify the crop rotation, to improve the soil quality and take advantage of symbiotic nitrogen fixation in the nodules of soybean roots^3^. High input costs and public pressure to reduce nitrogen use in agriculture in order to reduce environmental contamination increased the incentive to grow legumes. Soybean plants acquire nodulating bacteria, typically strains of the species *Bradyrhizobium japonicum*^4^ or *Bradyrhizobium diazoefficiens*^5^, from their rhizosphere by specific signalling, and maintain an intimate interaction with the symbionts^6^. The plant controls the nutrient supply and the number of nodules, and can induce senescence of nodules, to balance burden and benefit of the symbiosis^7^. Farmers inoculate the soybean seeds with commercial products containing efficiently nodulating strains to secure high yields^8,9^.

However, yield stability is a major concern when growing soybean. This problem is partially caused by pests and diseases. In some years, plant parasitic nematodes decrease soybean yield by more than 30% without visible symptoms aboveground^10^. In a recent survey of German soybean fields, we showed that the root lesion nematodes *Pratylenchus penetrans*, *P. neglectus* and *P. crenatus* are the main biotic threat of soybean production in such temperate regions, while the main threats worldwide, namely *Heterodera glycines*, *Pratylenchus brachyurus* or *Meloidogyne incognita*^11^, were not detected^12^. Early studies reported that plant parasitic nematodes could severely interfere with the soybean-rhizobia symbiosis. Nodules were decreased in number and size^13,14^, or increased in number with reduced nodule size^14^ due to root invasion by *H. glycines*, *P. penetrans*, or *Meloidogyne hapla*. Nitrogen-fixing capacity as measured by the acetylene reduction assay was significantly reduced by *P. penetrans* in a phytotron experiment compared to plants without nematodes, but not in a greenhouse experiment^14^. These experiments were performed with high numbers of infective stages of the nematodes that do not reflect normal field densities. In addition, the methods for the measurement of nitrogenase activity by the acetylene reduction assay that were applied in these studies can be highly inaccurate in predicting the rate of nitrogen fixation in nodules^15,16^. Thus, the density-dependent effects of root-lesion nematodes on the symbiosis of soybean plants with inoculants of *B. japonicum* are still unclear, and whether the nodule formation or the functioning of established nodules is affected by the nematodes. Systemic control of nodulation by the plant^17^ suggested that the intimate relationship is affected by systemic changes in the plant that are induced by root invasion of lesion nematodes. However, it was occasionally observed that some plant parasitic nematodes fed and reproduced in nodules^18,19^, which suggested a local mechanism of damage to the nodules.

The objectives of this study were to investigate whether the root lesion nematode *P. penetrans* affects the symbiosis of soybean plants with the nitrogen-fixing bacteria, and how this depends on the population density of the nematode in soil and in the roots. We further studied the disturbance of the established symbiosis by invading nematodes, and the effect of lesion nematodes in the roots on nodule formation. We determined as a measure for nitrogen fixation rate the concentration of ureides in the leaves. Ureides are synthesized by soybean plants from fixed nitrogen for transport from the nodules to the shoot^20^. As we observed migration of the nematodes into the nodules, we investigated whether the detrimental effect of the nematodes on nodule functioning is caused by the local damage of the nodules, or by a systemic mechanism.

## Results

### Density dependent effects of *P. penetrans* on rhizobial nodulation

**Figure 1:**
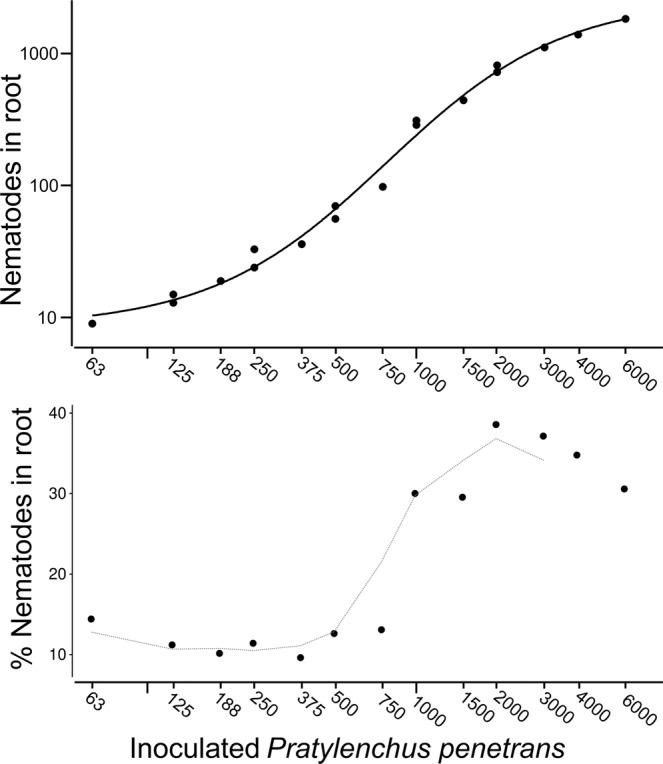
Density-dependent invasion of *Pratylenchus penetrans* into roots of soybean plants. Suspensions of infective stages of *P. penetrans* were equally inoculated around soybean plants into four holes of 5 cm depth, and invaded nematodes were stained and counted microscopically in the roots after two weeks. The numbers and percentages of nematodes that invaded the root system are shown relative to the number of nematodes that were inoculated to the plant two weeks before.

**Figure 2:**
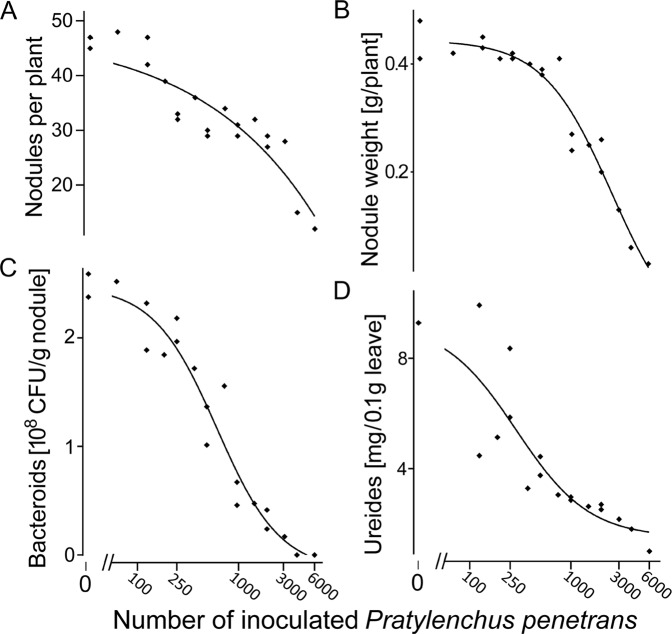
Density-dependent effects of *Pratylenchus penetrans* inoculated to soybean roots on nodule number (**A**), nodule weight (**B**), density of *Bradyrhizobium japonicum* bacteroids (**C**), and nitrogen fixation. (**D**) Viable bacteroids of a rifampicin resistant mutant of *B. japonicum* 532 C were extracted from 50 mg of nodules and quantified as colony forming units on selective yeast-mannitol agar plates containing vancomycin and rifampicin. Ureides (the transport forms of fixed nitrogen in soybean plants) were determined as allantoin and allantoic acid in 25 mg leaf tissue by a colorimetric assay. Curve fits are based on polynomal regressions using the software PRISM 7.

**Figure 3:**
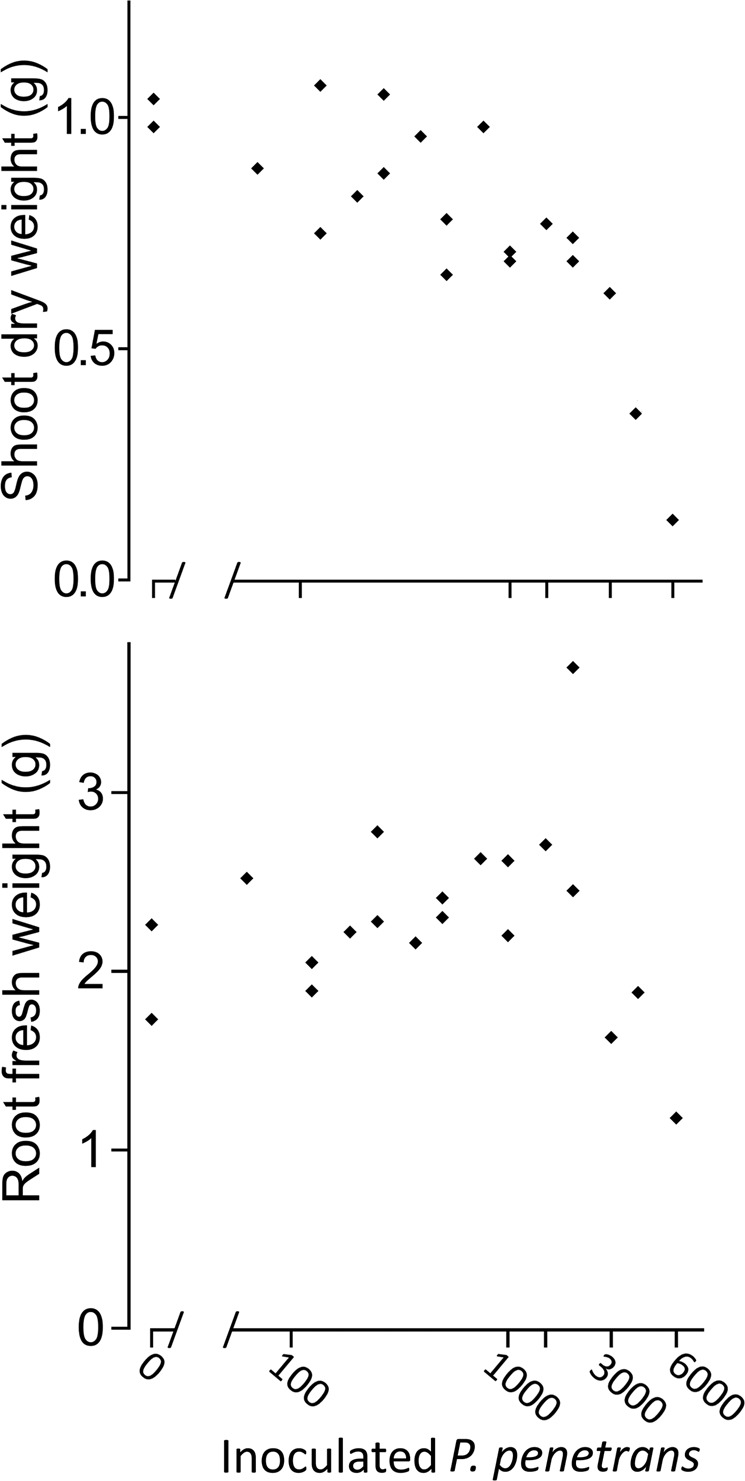
Density-dependent effects of *Pratylenchus penetrans* on growth of soybean plants. Root and shoot weights were determined five weeks after inoculation of *P. penetrans* and *B. japonicum* to the roots.

The roots of soybean were co-inoculated with *B. japonicum* and various numbers of *P. penetrans* to investigate density dependent effects of the nematodes on nodulation, symbiotic N_2_ fixation, and plant growth. The nematodes invaded the roots in a density dependent manner (Fig. 1). The percentage of inoculated nematodes that entered the roots slightly decreased from 14% with 60 inoculated nematodes to 10% with 375. There was a sharp increase above around 500 inoculated nematodes. With increasing numbers of nematodes in soil, the percentage of invading nematodes rapidly increased to 39%. Nematode counts in roots remained above 31% from 2,000 to 6,000 inoculated nematodes, despite the negative bias of counts at high density in roots. Increasing numbers of lesion nematodes progressively affected the number and weight of nodules per soybean plant, the number of viable bacteroids in the nodules, and the amount of fixed nitrogen measured as concentration of ureides in the leaves (Fig. 2). These negative effects, compared to the control plants without inoculated nematodes, started with about 250 infective nematodes. With 4,000 inoculated nematodes, hardly any viable bacteroids were detected and nodules developed only in small number and size. Ureides in the shoots (allantoin and allantoic acid) were near or even below detection limit, showing that no fixed nitrogen was transported to the leaves at these high infection rates. As a consequence of the nitrogen limitation, the shoot weight declined with increasing numbers of inoculated nematodes (Fig. 3A). The root reacted to low infestation by *P. penetrans* with slightly increased growth, while 3,000 and more inoculated nematodes affected the root weight (Fig. 3B). To estimate the threshold densities of inoculated *P. penetrans* above which the symbiosis was affected, the data were fit to the Seinhorst equation (Table 1). The shoot and root weight was affected above 250 or 2,000 nematodes, respectively. However, bacteroids, nodules, or ureides were affected at much lower densities above 0 to 130 inoculated *P. penetrans*.

**Table 1:** Estimation of threshold densities (T) of inoculated *Pratylenchus penetrans* (P_i_) above which the measured parameter of soybean plants or nodules was affected, using best fits to the Seinhorst equation^a^.

Parameter y	T	y_m_	y_m_ m	R²
Shoot weight	250	0.92	0.00	0.76
Root weight	2000	2.4	1.42	0.36
Bacteroids	30	2485	47	0.95
Nodule weight	130	0.44	0.004	0.94
Number of nodules	0	46	23	0.74
Ureides	0	22.9	6.3	0.75

^a^y = y_m_ m + y_m_ (1 − m) z^(Pi -T)^, for P_i_ > T; y = y_m_, for P_i_ ≤ T; y_m_ = y(P_i_ < T); y_m_ m = y(high P_i_); z = slope-determining parameter with 0.997 ≤ z < 1.

### Effect of *P. penetrans* on already nodulated soybean plants

**Figure 4:**
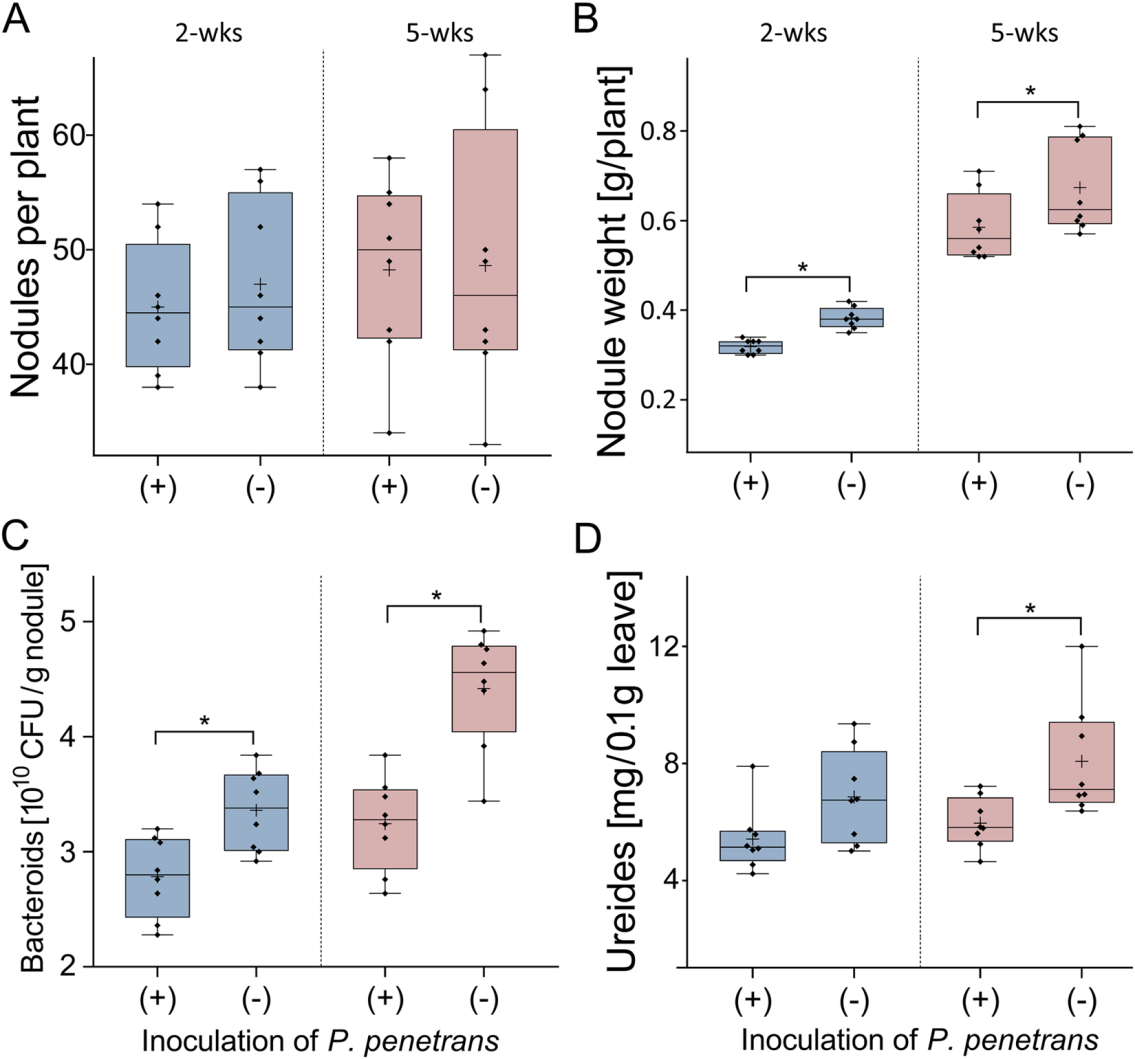
Effects of *Pratylenchus penetrans* on nodules and nitrogen fixation of soybean plants, which already established symbioses with *Bradyrhizobium japonicum*. Twelve-day old soybean seedlings were inoculated with *B. japonicum* and kept for two weeks until nodules have been fully established before inoculation of 1,000 *P. penetrans*. Two and five weeks thereafter, the number (**A**) and weight (**B**) of nodules, the density of viable bacteroids in nodules (**C**), and the concentration of transported fixed N_2_ in leaves (**D**) were determined. Significant differences between plants with and without inoculated nematodes are indicated by stars (Tukey’s test, n = 8, P < 0.05). Boxes indicate lower and upper quartiles separated by the median. Means are shown as (+). Whiskers extend to minimum and maximum values.

To investigate how an established *B. japonicum*-soybean symbiosis will be affected by invading lesion nematodes, soybean plants were first inoculated with *B. japonicum* and allowed to form nodules. Two weeks later, the nodulated plants were infected with 1,000 *P. penetrans* each. The effect of the root invasion of *P. penetrans* on the nodules and N_2_ fixation was analyzed two and five weeks after incubation of *P. penetrans*. The number of nodules did not significantly differ between the treatment with or without *P. penetrans*, and it did not change significantly over time (Fig. 4A). The mass of the nodules increased over time in both treatments, but was significantly greater in the non-infested control at both time points (Fig. 4B). The density of viable bacteroids in nodules increased over time and was significantly affected by *P. penetrans* two and five weeks after inoculation (Fig. 4C). Concomitantly, the transport forms of fixed nitrogen, the ureides, increased in concentration in the leaves over time. The concentration of ureides was significantly affected by *P. penetrans* at the 5-weeks sampling, and this trend was also visible at the 2-weeks sampling (Fig. 4D). In this experiment, the duration of treatments was not long enough to lead to a clear effect of *P. penetrans* on the growth of nodulated soybean plants (Supplementary Table S1). The shoot fresh weight was significantly decreased two weeks after incubation of *P. penetrans* compared to non-infected plants, and root weights showed the same trend.

### Effect of established *P. penetrans* on nodulation of soybean roots

**Figure 5:**
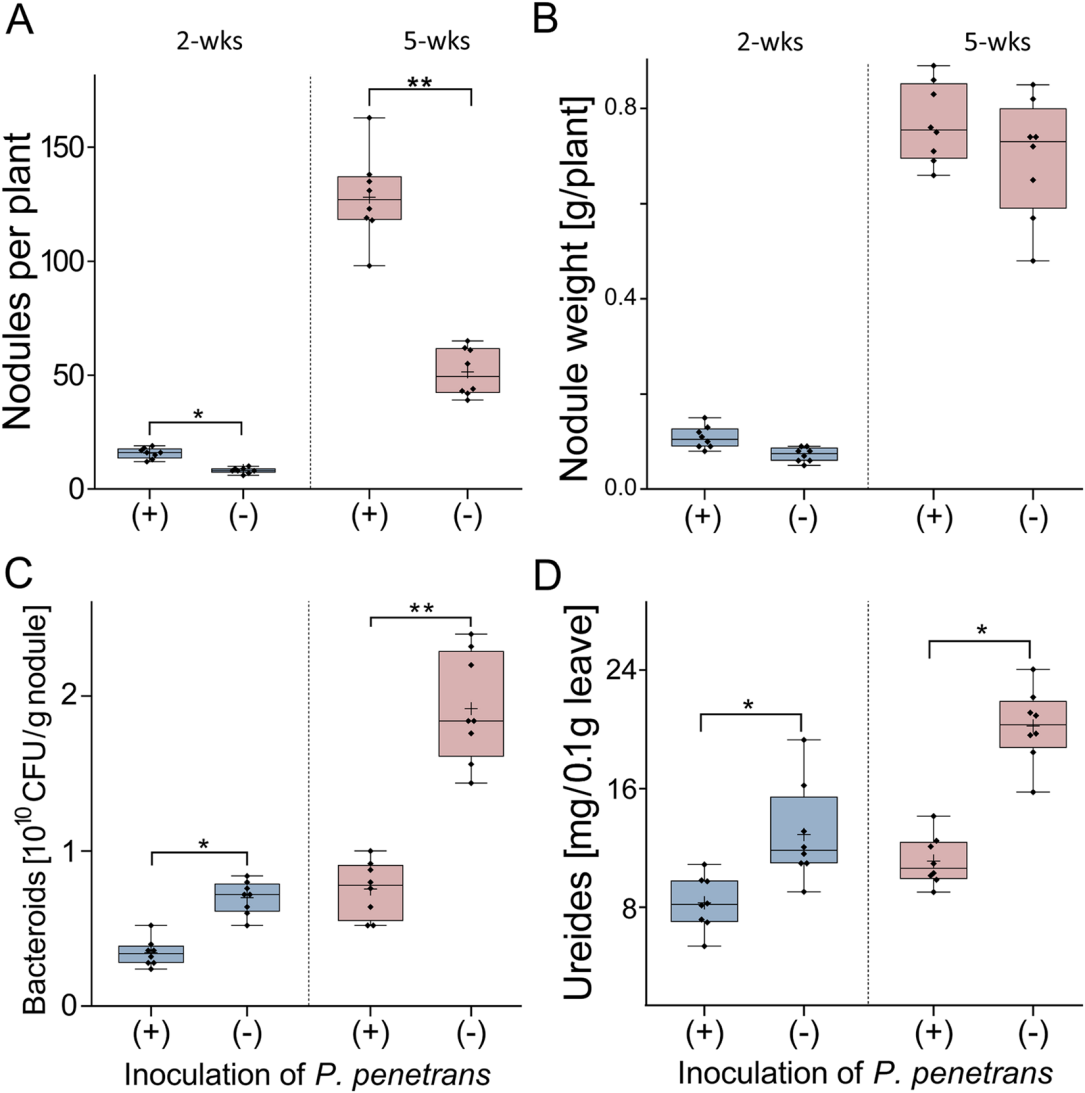
Effects of *Pratylenchus penetrans* already residing in roots on nodulation of soybean plants by *Bradyrhizobium japonicum*. The 12-day old soybean seedlings were infected with 1,000 *P. penetrans*. Two weeks later, pots were inoculated with *B. japonicum*. Two and five weeks after inoculation with *B. japonicum*, the number (**A**) and weight (**B**) of nodules, the density of viable bacteroids in nodules (**C**), and the concentration of transported fixed N_2_ in leaves (**D**) were determined. Significant differences between plants with and without inoculated nematodes are indicated by stars (Tukey’s test, n = 8, *P < 0.05, **P < 0.01). Boxes indicate lower and upper quartiles separated by the median. Means are shown as (+). Whiskers extend to minimum and maximum values.

To investigate how an established infestation with *P. penetrans* affects the nodule formation and function by *B. japonicum* in the soybean roots, the nitrogen fixing bacteria were inoculated to soybean roots that were already colonized by *P. penetrans*, or non-infected roots. Surprisingly, the infested roots formed a significantly higher number of nodules than the non-infested roots in the two weeks after inoculation of *B. japonicum* (Fig. 5A). Five weeks after inoculation of *B. japonicum*, the number of nodules even increased to an extreme value of 128 nodules on average per infested root, while the control plants had an average number of 51 nodules per root. The size of the nodules and the distribution across the root hairs clearly differed between the pre-infected and control roots. In the pre-infected soybean roots, where *P. penetrans* caused distributed lesions within the roots, the size of nodules was very small and clustered abnormally. In contrast, the nodules were large and well distributed across the root hairs in non-infested soybean roots. The higher number of nodules formed on the pre-infected roots was reflected by a slight trend for increased nodule mass, two and five weeks after inoculation of *B. japonicum* (Fig. 5B). However, the pre-infection of soybean roots with nematodes led to a significant decrease of viable bacteroids in nodules compared to the non-infested roots, two and especially five weeks after inoculation of *B. japonicum* (Fig. 5C). Consequently, ureide concentrations in leaves of infested plants were significantly lower than in leaves of non-infested plants at both samplings. Plant growth parameters did not significantly differ in this experiment where plants were grown with fertilizer before inoculation of the nodulating bacteria (Supplementary Table S2). The Supplementary Fig. S1 shows the change in root and nodule morphology caused by *P. penetrans*. Rhizobial colonization of soybean roots, measured as number of nodules per root mass, was strongly affected by the number of inoculated root lesion nematodes (Supplementary Fig. S2).

### Plant-mediated effect of *P. penetrans* on the *B. japonicum* - soybean symbiosis analyzed in split-root systems

**Figure 6:**
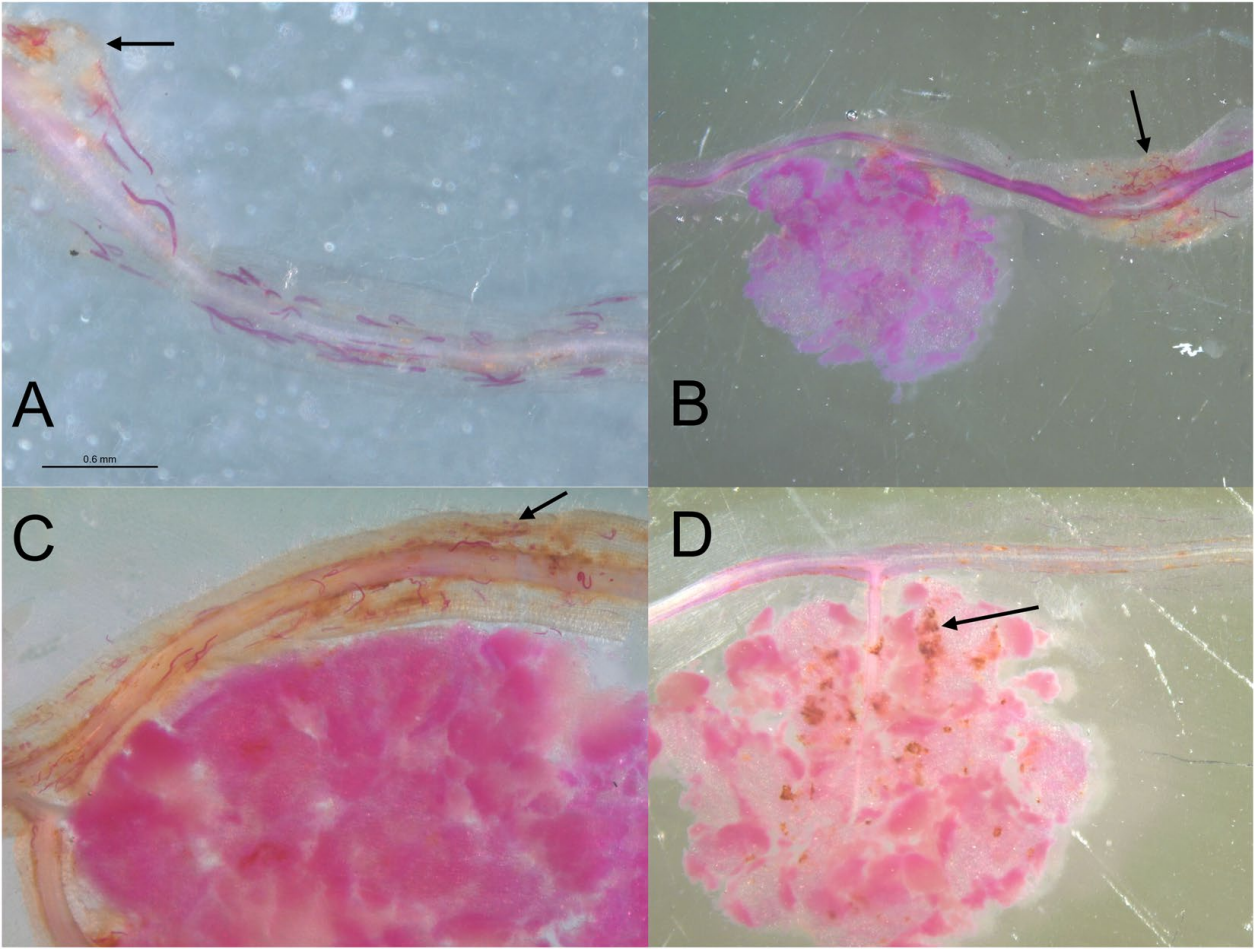
Colonization of nodules in soybean roots by *Pratylenchus penetrans*. (**A**) *P. penetrans* invaded the tissues of nodules and the area near to nodules. The nematodes were stained red with acid fuchsin. The arrow indicates a nodule with penetrated nematodes. (**B**) Eggs of *P*. *penetrans* laid near a nodule, indicated by the arrow. (**C**) *P. penetrans* damaged the cortex and phloem near to nodules. The arrow indicates lesions. (**D**) Damage to nodules by lesions (arrow) from migrating *P. penetrans*.

**Figure 7:**
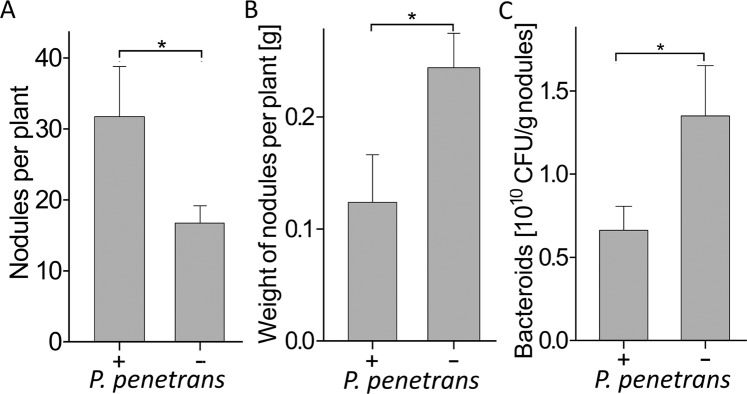
Systemic effect of *Pratylenchus penetrans* already residing in roots on the establishment of the symbiosis of soybean and *Bradyrhizobium japonicum*. In a split-root experiment, one-half of each root system was inoculated with 500 *P. penetrans* while control plants were not infected. After two weeks, the other half of all root systems was inoculated with *B. japonicum*. Three weeks after inoculation of *B. japonicum*, the number (**A**) and weight (**B**) of nodules per plant and the density of bacteroids (**C**) were analyzed. Significant differences between plants with and without inoculated nematodes are indicated by stars (Tukey’s test, n = 6, P < 0.05).

Microscopic analysis of nodules and areas near to nodules showed that *P. penetrans* invaded the nodules and the area close to nodules (Fig. 6A). They laid their eggs in such zones, suggesting that nodulation zones are a favorable habitat for these nematodes (Fig. 6B). The presence of *P. penetrans* within and close to nodules resulted in brown lesions within nodules and in the cortex and phloem near nodules (Fig. 6C,D). This looked like a direct effect of the nematodes on the functioning of the nodules. Therefore, we tested in a split-root system whether the effect of *P. penetrans* on the bacteria-plant symbiosis was partially plant-mediated by spatially separating nematodes from nodules. One side of the root system was either inoculated with *P. penetrans*, or kept as non-inoculated control. Two weeks later, the other half of the root system was inoculated with *B. japonicum*, and the roots were sampled after three weeks. As in the previous experiment, the number of nodules was significantly increased in roots of infested plants compared to the non-infested control (Fig. 7A). Nematodes were not able to migrate to the nodules in the other root system. The mass of nodules and the density of bacteroids was significantly decreased in infested plants compared to the non-infested control (Fig. 7B,C).

## Discussion

### Density dependent effects of *P. penetrans* on rhizobial nodulation

In this study, root invasion by *P. penetrans* affected the formation and development of nodules and thereby the N_2_ fixation by the *B. japonicum*-soybean symbiosis in a density-dependent manner. The effect on nodulation and N_2_ fixation was tested with a range of densities of lesion nematodes that reflected densities observed in the field, as well as with densities of 4,000 and more infective stages per plant that are unusually high at least during the early developmental period of soybean plants^12^. Other studies reporting effects of *P. penetrans*^14^ or *Heterodera glycines*^21^ on soybean nodules only applied very high densities that were largely above damage thresholds. This extreme number of nematodes resulted in severe effects on nodulation, nitrogen fixation and plant growth. In general, these early findings are in line with our results. However, the effect of *P. penetrans* on nitrogen fixation was not clearly shown in the earlier studies. The applied acetylene-ethylene assay suggested only a 19% reduction in the nitrogen fixing capacity of nodules compared to a control without *P. penetrans* in a phytotron experiment, and no reduction in a greenhouse experiment^14^. This underestimation of the effects of *P. penetrans* can be explained by inaccuracy of the assay as it was applied, and because the capacity of nitrogenase activity measured by this assay does not well reflect the amount of fixed nitrogen^15,16^. The ureides allantoin and allantoic acid are synthesized in the nodules of of tropical legumes, such as soybean and *Phaseolus vulgaris*, while asparagine and glutamine are the final products of nitrogen fixation in temperate legumes, such as pea and Faba bean^20^. In our study, we used a differential-colorimetric method^22^ to determine allantoin and allantoic acid that were synthesized with fixed atmospheric nitrogen in the nodules, and transported to the shoot tissues^23,24^.

The nodulation traits like nodule numbers and mass were negatively correlated with the density of *P. penetrans*. This might be explained by the competition between parasitic nematodes and rhizobial cells for resources provided by the host plant for the establishment of mutualism^25,26^. The soybean roots infected with higher densities of *P. penetrans* had significantly reduced nitrogen-fixing activity in the nodules as indicated by lower densities of bacteroids and less production of ureides compared to roots infected with lower numbers of nematodes, or uninfected plants. Migration of root lesion nematodes through the roots resulted in a destructive damage of root cells. The basal defense of the plant led to further damage of the root tissue. This might have interrupted the flux and delivery of nutrients that the plant provides to support the bacterial symbiont^27,28^. In addition, pathogen induced defense can negatively affect the mutualistic relationship as shown for both rhizobia and arbuscular mycorrhizal fungi^29^.

Our data demonstrated that high densities of *P. penetrans* reduced the shoot dry mass. The root reacted to low infestation by additional growth at low infection rates, as the plant probably tries to compensate for damaged tissue to sustain nutrient and water uptake. High numbers of 1,000 and more infective stages invading the root significantly affected the root weight. It is apparent that the relation between density and plant growth is complicated^30^. In some cases, damage by nematodes to the root does not result in the reduction of shoot weight because plants may have more roots than needed to support the shoot, or plants compensate the damage to roots by building more side roots. However, their potential to compensate the damage is determined by the availability of nutrients.

### Effect of nematodes on the maturation of nodules

We showed that the order of establishment and colonization of roots by *P. penetrans* and *B. japonicum* largely determined the effects on the nodules. After initial colonization of roots by rhizobial cells and establishment of the nodules, *P. penetrans* infection had no effect on the number of nodules compared to non infected roots. Probably the acquisition of *B. japonicum* by the roots had already been completed. However, the further maturation of the nodules was impaired, as indicated by the slower increase in the weight of nodules and density of bacteroids within the nodules. Both, mutualistic and pathogenic partners induce significant changes in phytohormone levels^31^. With rhizobia, this leads to transportation of the fixed nitrogen and in turn enhances the flux of carbon and amino acids to the bacteroids^32^. This nutrient fluxes can be affected by complex changes in the vascular tissues, sugars transport and phytohormones regulation, that in turn affect the growth of nodules and survival of bacteroids^33,34^. In addition, the invasion of nematodes can affect the viability and differentiation of bacteroids by reducing the availability of leghemoglobin, which regulates the supply of oxygen to protect the rhizobial nitrogenase. Accordingly, the concentration of ureides in the leaves was significantly lower in plants infested by *P. penetrans*.

### Interference of lesion nematodes with the regulation of nodulation

Interestingly, *P. penetrans* that was already established in roots had a very severe impact on nodulation by *B. japonicum*. A dramatically higher number of nodules developed on infested roots compared to the non-infested roots. This hyper-nodulation resulted in small-sized and aggregated nodules containing low densities of bacteroids. The majority of these clustered small nodules were formed near to the damaged crown of the roots. Nodulation of legume roots is a resource demanding process and therefore tightly regulated^17^. This autoregulation seemed to be disturbed in the infested roots. The nodulation is regulated through systemic mechanisms where the plant coordinates the nodulation and suppresses further formation of nodules by signaling from shoot to root and back again^6,35^. We showed in the split-root experiment that the nematodes had a systemic effect on the nodulation, but local damage to the nodules was also observed. Lesions formed by *P. penetrans* near the nodules releases extracellular ATP, which acts as a damage-associated molecular patterns but likely plays a role in the regulation of nodulation as well^6^, which might result in a local interference of *P. penetrans* and the soybean - *B. japonicum* symbiosis. Another key to this interaction might be cytokinin, because cytokinin signaling is important for the control of nodulation by legumes^7^, and cytokinin was also reported to play a role in the parasitism of root knot and cyst nematodes, which produce cytokinin and manipulate cytokinin signaling of the host plant^36^. A local excess of cytokinin likely leads to hyper-nodulation because it acts as an endogenous inducer of nodule primordia formation^7^. However, cytokinin production was not yet shown for root lesion nematodes^27^. In our study, aggregated hyper-nodulation was observed on locally infested roots but much less in the split-root system where *P. penetrans* was spatially separated from nodules. A similar effect was achieved by removal of nodules, which was suggested as an indication for a locally generated signal of autoregulation of nodulation^37^.The damage of nodules by the migrating nematodes might result in a new round of nodulation by the same mechanism.

It was previously reported that the number of nodules per plant was not well correlated with the total mass of nodules per plant and the amount of fixed nitrogen^38^. It seems that the plant initiated more nodulation processes but the nodules did not become mature. In a *Phaseolus vulgaris* - *Rhizobium leguminosarum* symbiosis, the number of nodules was also systemically affected by a fungal leaf pathogen that was inoculated in the initial phase of nodule formation by the nitrogen fixing symbiont, but in contrast to our study the number of nodules was significantly reduced^29^. The fungal pathogen systemically induced higher activity of polyphenol oxidases in the roots, suggesting that plant defense responses interfered with nodulation. In another study, chemically induced defense pathways in soybean systemically reduced the number of nodules and the nitrogen content in leaves and roots after 51 days^39^. The chemical inducer was applied once as foliar spray 5 days after inoculation of seedlings with *B. japonicum*, while *P. penetrans* could continuously affect the soybean - *B. japonicum* symbiosis. In our study, the small sized nodules from pre-infested roots had very low densities of bacteroids compared to those recovered from healthy roots. Limitation of O_2_ supply for the bacteroids in the nodules as a response of the plant to stress caused by the nematodes could explain the reduced viability of bacteroids^40^. This was also evidenced by a significant decline in the ureides amount in the infected plants.

In sum, results contribute to a better understanding of the interaction between soybean plants, nitrogen fixing bacteria and *Pratylenchus* that might lead to strategies to improve N_2_ fixation under pathogen pressure. Early nodulation before the nematode population builds up, and control of population densities of root lesion nematodes are important for harnessing the positive effects of symbiotic nitrogen fixation. In the future, it will be of interest to expand our investigation to explore the spatial distribution of plant parasitic nematodes within the nodulated roots and to understand the reasons behind the preference of nematodes to migrate and propagate near to nodule formation zones.

## Materials and Methods

### Soybean cultivation

Seeds from soybean cv. Primus were surface sterilized in 1.5% sodium hypochlorite for 15 min and rinsed with sterile distilled water. Seeds were transferred on moist sterilized filter paper in Petri dishes placed in the dark at 20 °C for 4 days to allow germination. Uniformly germinated seedlings were selected and transplanted in 12 cm diameter plastic pots filled with 500 ml growth substrate. Except for the split-root experiment, the growth substrate consisted of two volumes of sand and one volume of a low nitrogen content field soil (7.2 kg N_min_ per ha, loamy sand, braunerde, pH 6.5, 52°17′57″N/10°26′14″E)^41^. Plants were maintained in the greenhouse at 24 °C and 16 h photoperiod and watered as needed every 2–3 days.

### *B. japonicum* inoculum

An isolate of strain *B. japonicum* 532C was recovered from HiStick Soy (BASF, Ludwigshafen, Germany) by plating the product on a yeast mannitol (YM) agar supplemented with 1 mg/l vancomycin. To support the axenic recovery of the strain from nodules, a rifampicin resistant mutant was isolated. This was done by plating a high-density culture on YM supplemented with 1 mg/ml vancomycin^42^ and 50 mg/l rifampicin, and purifying streaks of a single rifampicin-resistant colony on these agar plates. The *B. japonicum* strain was cultured for 3 days at 28 °C on YM liquid medium supplemented with rifampicin. The *B. japonicum* cells were spun down at 4,000 g for 10 minutes, and the pellet was washed twice with sterile tap water to remove antibiotic residues, resuspended in sterile tap water and adjusted to OD_600_ = 0.2. In the greenhouse experiments, each pot was inoculated with 4 ml of the *B. japonicum* cell suspension by adding it to four holes of 5 cm depth around the soybean root.

### *P. penetrans* inoculum

The root lesion nematode *P. penetrans* was extracted from 4 months old carrot disks by the Baermann funnel technique^43^. The densities of nematodes in 1 ml of the suspensions were microscopically determined in a nematode-counting slide. The nematode suspension of mixed infective stages of *P. penetrans* was equally inoculated around soybean plants into four holes of 5 cm depth. The number of *P. penetrans* inoculated differed based on the experimental layout.

### Greenhouse experiment on density-dependent effects of *P. penetrans* on nitrogen fixation by co-inoculated *B. japonicum*

Inocula of *P. penetrans* were prepared by serial dilution of three independently extracted suspensions of the nematodes. One week after germination, soybean plants were infected with 0, 0, 63, 125, 125, 188, 250, 250, 375, 500, 500, 750, 1000, 1000, 1500, 2000, 2000, 3000, 4000, or 6000 individuals of *P. penetrans*. Each of the 20 plants was co-inoculated with *B. japonicum* and randomly distributed in a greenhouse chamber. One month after inoculation, the root weight, shoot weight, concentration of ureides in leaves, number of nematodes in roots, and number and weight of nodules were determined.

### Greenhouse experiment on effects of *P. penetrans* on established nodules

Twelve-day-old soybean seedlings were inoculated with *B. japonicum* and kept for two weeks until nodules have been established. Sixteen nodulated plants were inoculated with 1,000 infective stages of *P. penetrans*. Sixteen control plants were not infected with nematodes. Plants were randomly distributed in a greenhouse chamber. Two and five weeks after inoculation, eight plants per treatment and time point were sampled to determine the root weight, shoot weight, ureide concentration in leaves, number of nematodes in roots, and number and weight of nodules.

### Greenhouse experiment on effects of established *P. penetrans* on nodulation

This experiment was implemented to investigate how the process of nodulation and N_2_ fixation is affected when the roots are infested by *P. penetrans* before inoculation of *B. japonicum*. Sixteen 12-day-old soybean seedlings were infected with 1,000 *P. penetrans*. Sixteen control plants were not infected with nematodes. Plants were randomly distributed in a greenhouse chamber. Two weeks later, all pots were inoculated with *B. japonicum*. Eight plants per treatment and time point were sampled two weeks and five weeks after inoculation with *B. japonicum* to determine the root weight, shoot weight, ureide concentration in leaves, number of nematodes in roots, and number and weight of nodules.

### Split-root experiment on plant-mediated effects of *P. penetrans* on the nodulation by *B. japonicum*-soybean symbiosis

The root of twelve two-week-old soybean seedlings was split and transplanted into two adjacent square pots (7 cm × 7 cm) filled with two times autoclaved sand. For half of the plants, the split-root system in one pot was inoculated with 500 *P. penetrans*, the other plants were non-inoculated controls. After two weeks, one-half of each root system without nematodes was inoculated with 2 ml of a *B. japonicum* suspension (OD_600_ = 0.2). The plants were watered and fertilized by a mineral solution^44^ supplemented with 0.125 mM NH_4_NO_3_. Three weeks after inoculation of *B. japonicum*, the plants were sampled to determine the root weight, shoot weight, number of nematodes in roots, and number and weight of nodules.

### Analysis of plant samples

The numbers of viable bacteroids of *B. japonicum* per 0.05 g nodules (3–7 nodules) were determined by surface sterilizing the nodules for 10 min in 1% sodium hypochlorite, washing them with sterilized water, squeezing with the tip of a pipette in 2 ml microtubes, vortexing in 1 ml sterile 0.85% NaCl for 15 s, and plating serial dilutions of the homogenate onto YM agar supplemented with 1 mg/l vancomycin and 50 mg/l rifampicin. Colony forming units (CFU) were counted after incubation of the plates for three days at 28 °C.

The concentration of transported ureides in the leaves was determined according to the protocol of Collier and Tegeder^20^. Briefly, 0.2 g leave tissue was frozen in liquid nitrogen and stored at −80 °C until analysis. The tissue was ground with mortar and pestle and the powder was transferred to 2 ml microtubes with 200 µl of ice-cold sterile deionized water, squeezed with a plastic micro pestle, which was carefully rinsed off with 800 µl sterile H_2_O. The suspension was filtered through a layer of Miracloth (Merck, Darmstadt, Germany) into 1.5 ml microtubes and centrifuged at 20,000 g at 4 °C for 30 minutes. To measure the concentrations of total ureides and allantoic acid, two colorimetric assays were performed using phenylhydrazine and potassium ferricyanide as reaction reagents and 100 µl of the filtrate for each reaction. The absorbance at 520 nm was measured within 30 minutes using a spectrophotometer, and compared to a standard curve of 0 mM, 1 mM, 10 mM, 50 mM, 100 mM, 200 mM, and 300 mM allantoin (Sigma-Aldrich, Munich, Germany). The allantoin concentration was calculated by subtracting the concentration of allantoic acid from the concentration of total ureides.

The nematodes within the roots were stained with 1% acid fuchsin^45^. Sections of 3 cm roots were embedded in glycerol and several slides were prepared to count the nematodes. The microscopic analyses were performed with a stereomicroscope (Olympus Microscope SZX12) and photographed with a digital camera.

### Statistical analyses

Graphs were generated, and statistical tests on treatment effects were done using Prism 7 (GraphPad Software, La Jolla, CA, United States). Effects of inoculated nematodes on nodule number, nodule weight, bacteroid CFU, and ureide concentration were tested against the non-inoculated control for each analyzed sampling time using Tukey’s LSD test. To estimate the threshold densities of inoculated *P. penetrans* above which the shoot weight, root weight, number of viable bacteroids, number or weight of nodules, or concentration of ureides was affected, the data were fit to the Seinhorst equation^30^ using the program SeinFit^46^. An example of the output of the program is shown in Supplementary Fig. S3.

## Supplementary information

Supplement.

## Data Availability

All materials, data and associated protocols are available from the corresponding author on reasonable request.
