# Supplementary material for: Symbiosis of soybean with nitrogen fixing bacteria affected by root lesion nematodes in a density-dependent manner
Source: Sci Rep. 2020 Jan 31;10:1619. doi: 10.1038/s41598-020-58546-x (PMC6994534; doi:10.1038/s41598-020-58546-x)

Supplement

Symbiosis of soybean with nitrogen fixing bacteria affected by root lesion nematodes in a density-dependent manner

Ahmed Elhady[1,2], Johannes Hallmann[1], Holger Heuer[1]

[1] Department of Epidemiology and Pathogen Diagnostics, Julius Kühn-Institut, Federal Research Centre for Cultivated Plants, Braunschweig / Münster, Germany

[2] Department of Plant Protection, Faculty of Agriculture, Benha University, Benha, Egypt

Table S1. Effect of *Pratylenchus penetrans* on growth of fully nodulated soybean plants.

Plant weight [g]	5-weeks sampling *		8-weeks sampling	
	+ *P. penetrans*	- *P. penetrans*	+ *P. penetrans*	- *P. penetrans*
Root fresh weight	2.9 ± 0.3 a	3.6 ± 0.3 a	7.1 ± 0.9 a	7.7 ± 1.1 a
Shoot fresh weight	4.0 ± 0.4 b	4.4 ± 0.2 a	11.2 ± 0.8 a	11.1 ± 1.2 a
Shoot dry weight	0.8 ± 0.1 a	0.9 ± 0.0 a	2.6 ± 0.1 a	2.6 ± 0.3 a

* Different letters indicate significant differences among treatments within a sampling (Tukey's test)

Table S2. Effect of *Pratylenchus penetrans* on growth of soybean plants during nodulation by *Bradyrhizobium japonicum*.

Plant weight [g]	5-weeks sampling *		8-weeks sampling	
	+ *P. penetrans*	- *P. penetrans*	+ *P. penetrans*	- *P. penetrans*
Root fresh weight	2.5 ± 0.4 a	2.5 ± 0.3 a	7.1 ± 0.9 a	6.3 ± 0.6 a
Shoot fresh weight	3.5 ± 0.5 a	3.6 ± 0.3 a	8.9 ± 1.2 a	7.9 ± 1.3 a
Shoot dry weight	0.7 ± 0.1 a	0.8 ± 0.1 a	2.1 ± 0.4 a	2.1 ± 0.2 a

* Different letters indicate significant differences among treatments within a sampling (Tukey's test)

Figure S1. Nodules formed in soybean roots that were infested or non-infested by *Pratylenchus penetrans*. Soybean seedlings were inoculated with 1,000 infective stages of *P. penetrans*, and two weeks later inoculated with *Bradyrhizobium japonicum*. Nodulation in the following five weeks resulted in more and smaller nodules in the presence of the nematodes (left) compared to non-infested roots (right). *P. penetrans* also affected the root morphology.

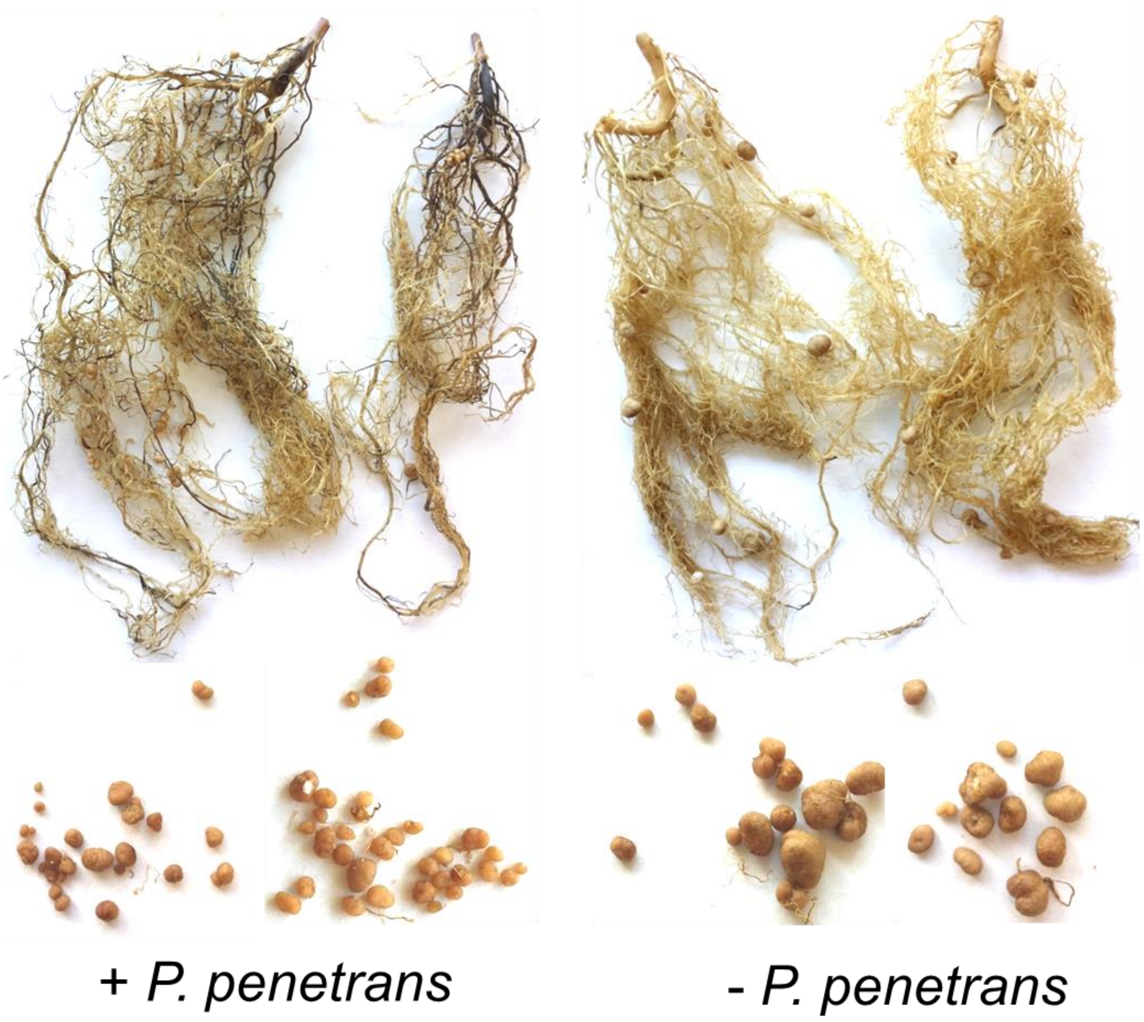

Figure S2. Negative correlation of the rhizobial colonization of soybean roots, measured as number of nodules per root mass, and the number of inoculated root lesion nematodes (log scale). The linear model (nodules per gram) = log(nematodes+1) was highly significant, as determined by SAS 9.4 proc REG.

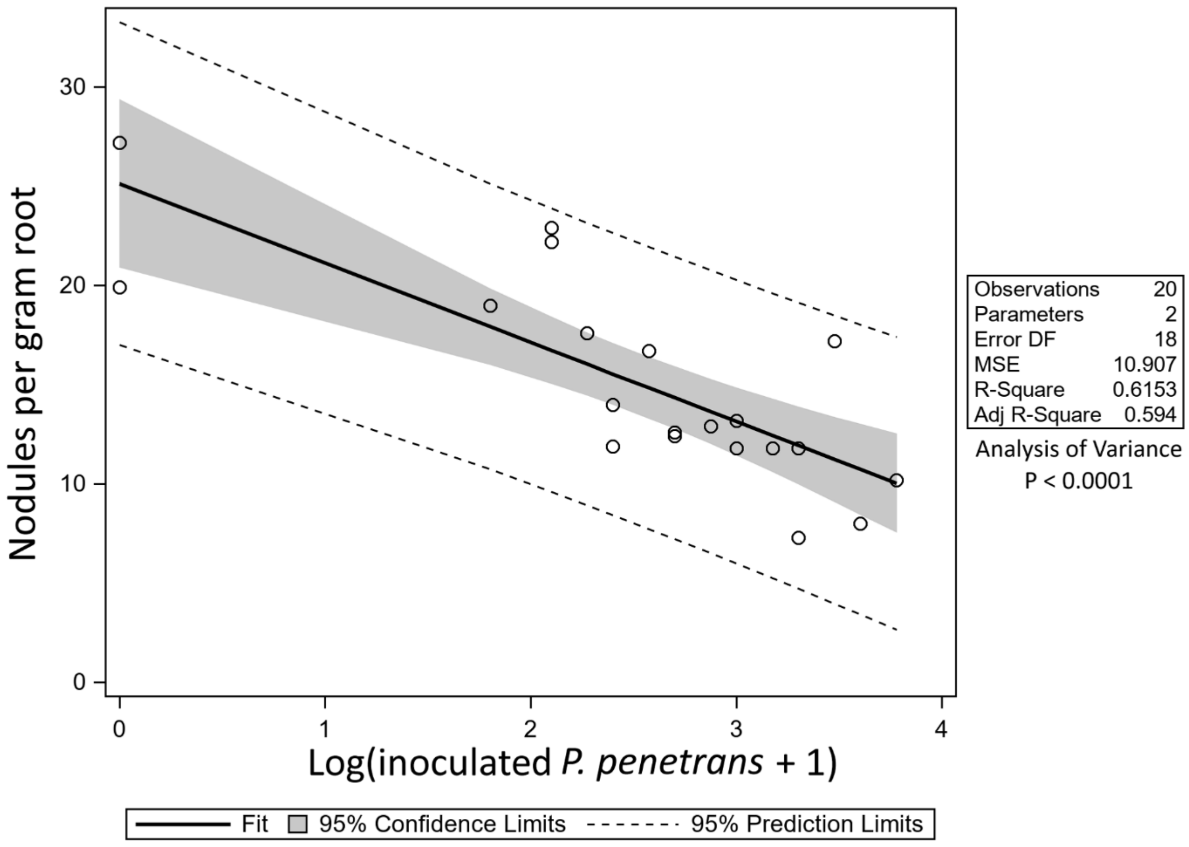

Figure S3. Estimation of threshold densities of inoculated *Pratylenchus penetrans* above which the number of viable bacteroids in nodules was affected, using best fits to the Seinhorst equation (Viaene, N. M., Simoens, P. & Abawi, G. S. 1997. *J. Nematol.* **29,** 474–477).

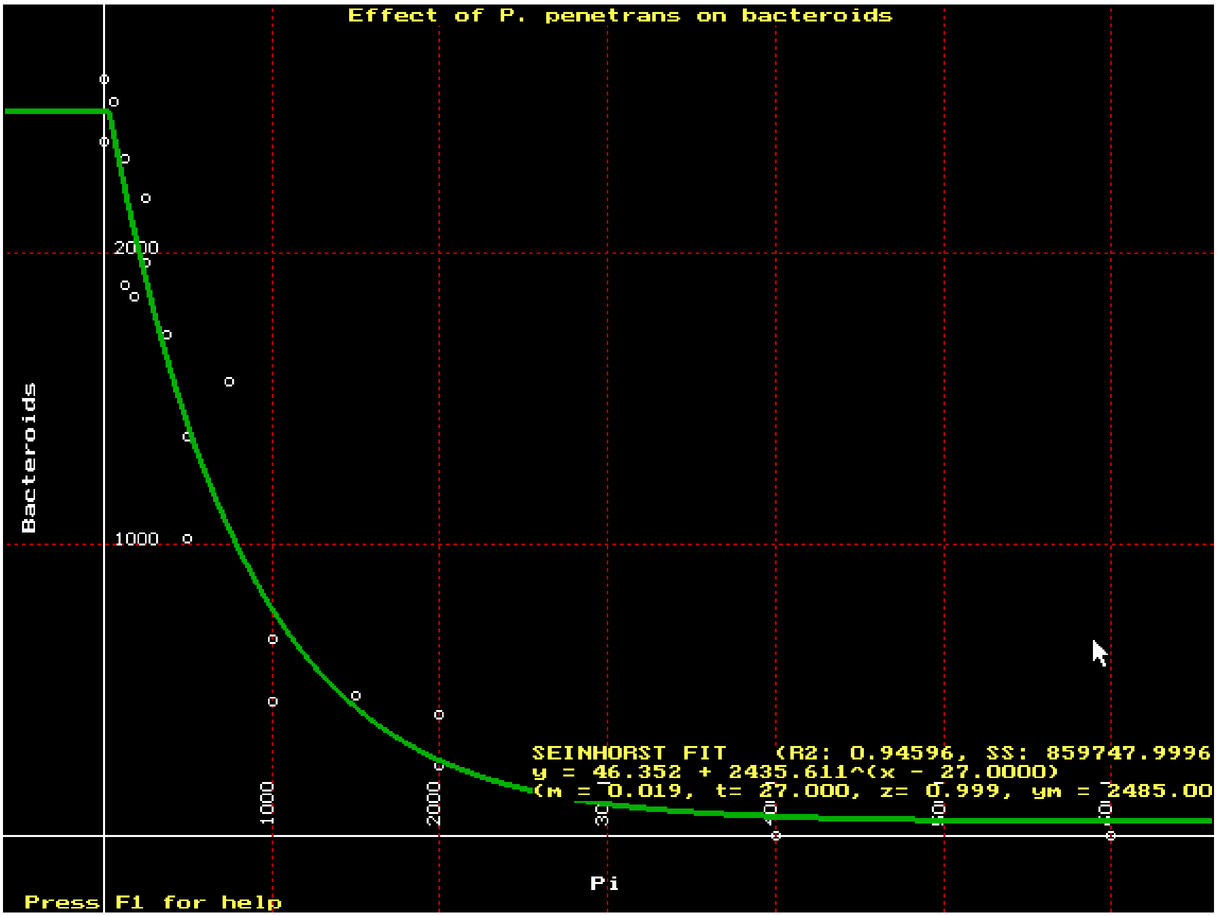

Supplement: Supplementary file 1 — Supplement. [file 41598_2020_58546_MOESM1_ESM.pdf]
